# Homozygous substitution of threonine 191 by proline in polymerase η causes Xeroderma pigmentosum variant

**DOI:** 10.1038/s41598-023-51120-1

**Published:** 2024-01-11

**Authors:** Roberto Ricciardiello, Giulia Forleo, Lina Cipolla, Geraldine van Winckel, Caterina Marconi, Thierry Nouspikel, Thanos D. Halazonetis, Omar Zgheib, Simone Sabbioneda

**Affiliations:** 1 Istituto di Genetica Molecolare “Luigi Luca Cavalli-Sforza”, CNR, Pavia, Italy; 2 Dipartimento di Biologia e Biotecnologie ‘Lazzaro Spallanzani’, Università degli Studi di Pavia, Pavia, Italy; 3 Division of Medical Genetics, Diagnostics Department, Geneva University Hospitals, Geneva, Switzerland; 4 Department of Molecular and Cellular Biology, University of Geneva, Geneva, Switzerland

**Keywords:** Genomic instability, Mechanisms of disease, Translesion synthesis, Translesion synthesis, Genetic testing

## Abstract

DNA polymerase eta (Polη) is the only translesion synthesis polymerase capable of error-free bypass of UV-induced cyclobutane pyrimidine dimers. A deficiency in Polη function is associated with the human disease Xeroderma pigmentosum variant (XPV). We hereby report the case of a 60-year-old woman known for XPV and carrying a Polη Thr191Pro variant in homozygosity. We further characterize the variant in vitro and in vivo, providing molecular evidence that the substitution abrogates polymerase activity and results in UV sensitivity through deficient damage bypass. This is the first functional molecular characterization of a missense variant of Polη, whose reported pathogenic variants have thus far been loss of function truncation or frameshift mutations. Our work allows the upgrading of Polη Thr191Pro from ‘variant of uncertain significance’ to ‘likely pathogenic mutant’, bearing direct impact on molecular diagnosis and genetic counseling. Furthermore, we have established a robust experimental approach that will allow a precise molecular analysis of further missense mutations possibly linked to XPV. Finally, it provides insight into critical Polη residues that may be targeted to develop small molecule inhibitors for cancer therapeutics.

## Introduction

Xeroderma pigmentosum variant (XPV), an autosomal recessive disease, was first described in 1970 as having a similar phenotype to xeroderma pigmentosum (XP), however with less severe sun sensitivity, freckling, neoplastic transformation and a later onset^1^. Its molecular basis also differs from that of XP, as translesion synthesis (TLS) and not nucleotide excision repair (NER) is defective in XPV. Complementation assays with HeLa cell isolates led to the identification in 1999 of DNA polymerase eta (Polη), highly conserved in eukaryotes, and capable of restoring replication of DNA containing cyclobutane pyrimidine dimers (CPD)^2^. Polη, which is constitutively present at the replication fork, enables efficient thymine-thymine (TT) dimer replication. It can also bypass, albeit less efficiently, other lesions such as O6-methylguanine (O6mG), 8-oxoguanine (8-oxoG), and cisplatin-induced guanine-guanine (GG) adducts^3^. Polη is the only TLS polymerase capable of error-free bypass of UV-induced CPD, and it was the first TLS polymerase associated with a human disease. The gene coding for Polη is *POLH* and contains 11 exons located on 6p21.1-6p12.3. The importance of TLS in human health is further strengthen by recent findings showing that mutations in *REV3L*, a gene encoding for another TLS polymerase, can cause Möbius syndrome^4^ and developmental delay with hypotrophy^5^.

The mechanism behind TLS involves monoubiquitylation of the Proliferating Cell Nuclear Antigen (PCNA), promoting its interaction with Polη and facilitating the polymerase switch^6^. The crystal structure of Polη′s catalytic domain was first solved in *S. cerevisiae* and it provided the basis for its unique function in faithfully replicating DNA containing a CPD. This capability is linked to its wider and more open active site that can accommodate more than one unpaired nucleotide^7^. On the other hand, this specific feature makes the polymerase more prone to errors when replicating undamaged DNA.

The structure of the catalytic domain of human Polη was solved in 2010 and it similarly features a large active site. The full protein is composed of 713-amino acids, with an N-terminal catalytic domain and a C-terminal region comprising the PCNA interacting region, a ubiquitin-binding domain (UBZ), a nuclear localization signal (NLS) and domains that bind REV1, another TLS polymerase, and POLD2, a subunit of the replicative Polδ polymerase^8–10^. The first 500 residues of Polη constitute the catalytic domain, whose structure, spanning residues 1–432, is composed of a palm (1–18, 88–239), finger (16–87), thumb (240–306) and little finger (307–432) domains^10^.

A search of the main public archives that report evidence-based relationships among human variations and phenotypes, namely Human Gene Mutation Database (HGMD), Leiden Open Variation Database (LOVD), and NCBI’s ClinVar, shows that all reported pathogenic or likely pathogenic Polη variants are loss of function truncation or frameshift mutations, mainly in the catalytic domain. To date, no missense variant has been reported as being pathogenic or likely pathogenic, owing to the lack of evidence-based in vitro or in vivo functional studies adhering to the American College of Medical Genetics and Genomics (ACMG) guidelines for variant interpretation^11^.

A Thr191Pro variant was recently identified in Brazilian XPV patients and it was associated with skin tumors, namely squamous cell carcinoma (SCC). This variant, alongside others identified in the same study, was reported as of uncertain significance (VUS) due to the lack of functional data^12^.

Here we report the case of a 60-year-old woman carrying the Thr191Pro variant in homozygosity. We characterize Polη carrying the amino acid substitution in vitro and in vivo, providing molecular evidence that the substitution abrogates polymerase activity and results in UV sensitivity through deficient damage bypass.

## Results

**Figure 1:**
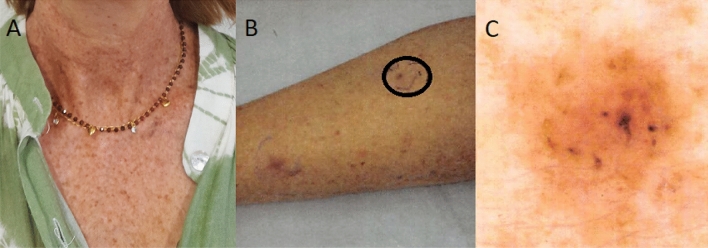
(**A**) Patient photographs showing freckles on neck and torso; (**B**) Patient’s leg with a close-up view (**C**) of a superficial basal cell carcinoma before excision.

The patient, who was referred to us for genetic counseling and testing, was known for XPV since her late teens. She presented initial freckling at age four and a first nose lesion at age 14. Recurrence after multiple excisions prompted a skin graft on her nose at age 24, at which time she started follow-ups at our university hospital. She later developed multiple squamous and basal cell carcinomas (BCC), as well as melanomas, mostly on the face and limbs. She also presented right renal atrophy and a small head circumference (52 cm, < P3) (Fig. 1A–C). There were no neurological symptoms and ophthalmological assessment was normal.

**Figure 2:**
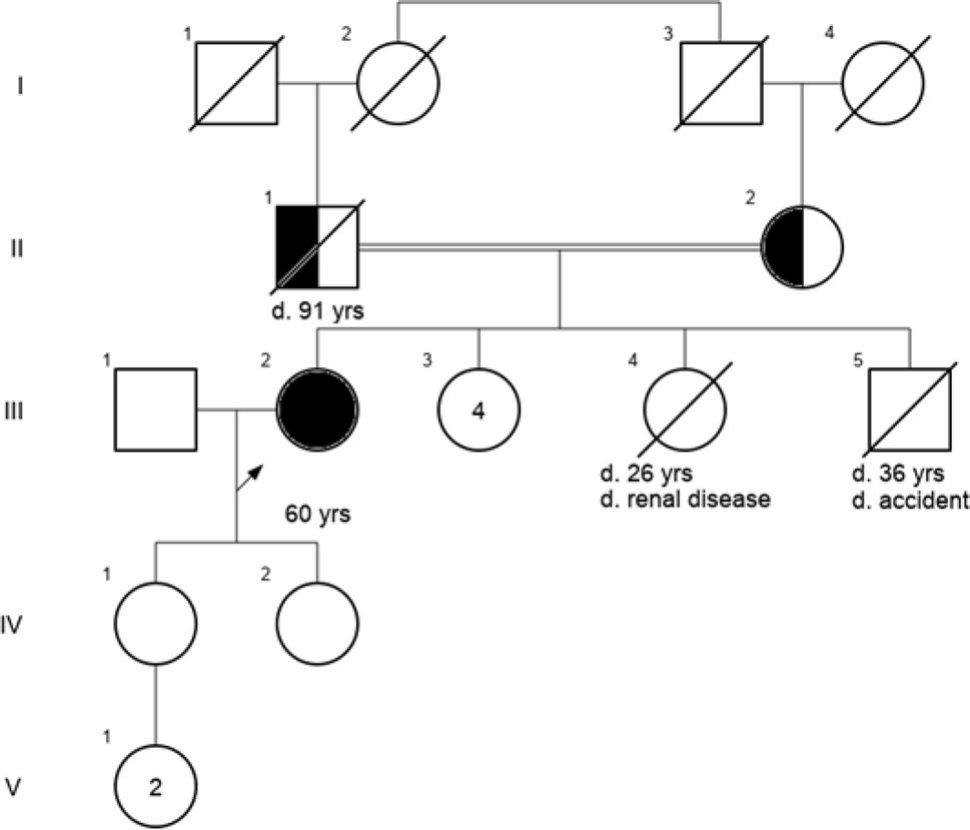
Patient’s pedigree.

Family history showed that her parents were first-degree cousins of Portuguese descent. She had six siblings, including a brother and sister who passed away in an accident at age 36 and from renal disease at age 26, respectively. Her four other sisters, one of whom was described as having freckles and a normal skin biopsy, were in good health. None of her siblings was known for skin cancer (Fig. 2).

In this setting of an inbred patient with XPV, we highly suspected a homozygous mutation in *POLH* and carried out targeted exome sequencing. We found no variants in a gene panel of XP, trichothiodystrophy, or Cockayne syndrome, except for one, at the homozygous state, in *POLH*: NM_006502:exon5:c.571A > C:p.Thr191Pro. In the absence of functional data, the variant was classified as of uncertain significance (VUS).

Our search of the literature found that it was reported for the first time in 2020 in XPV patients and indeed classified then as VUS^12^. Only the nucleotide substitution (c.571A > C) was reported in the body of the article, with the supplementary section indicating the resulting Thr191Pro substitution. Its predicted functional impact was not described, and no functional assays were performed.

**Figure 3:**
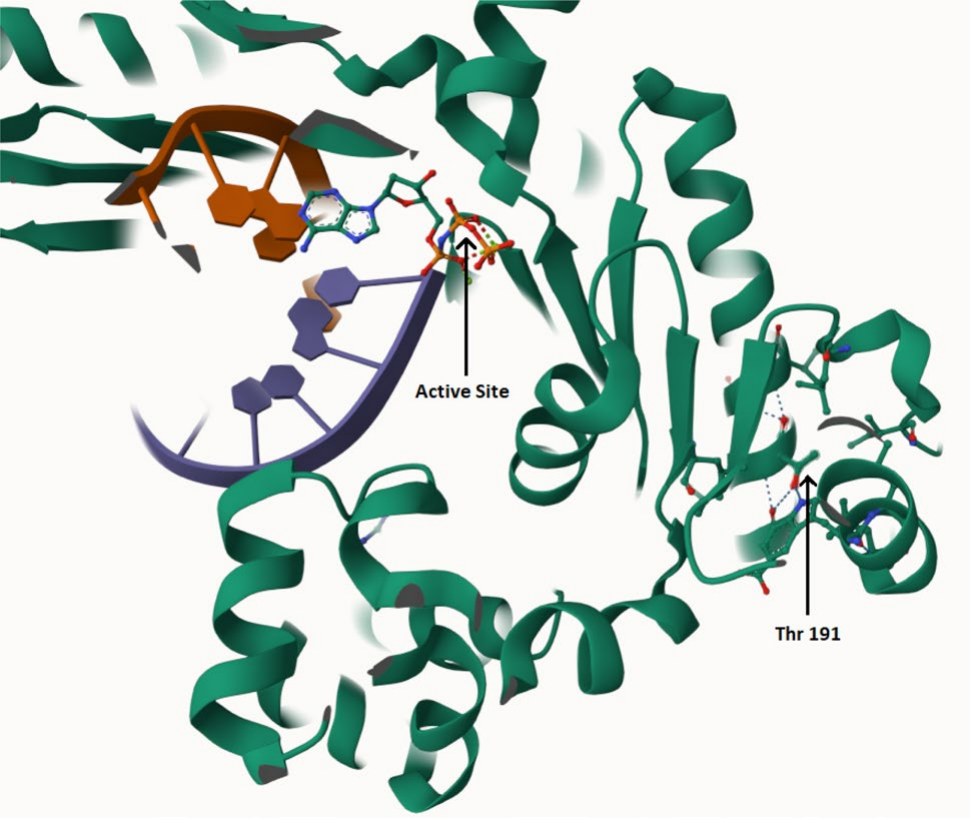
Mol* Viewer three-dimensional structure of Polη showing the catalytic domain with the DNA double strand and an incoming nucleotide in the active site. Thr191 is shown with stabilizing intra- and inter-helical interactions (see text for details).

Thr191 is located away from the nucleotide binding site, but in the midst of an alpha-helix in the palm domain, its hydroxyl sidechain making close contact with the sidechain nitrogen of Trp174, located in an opposite alpha-helix, and the backbone carboxyl of Asp187, four residues upstream. Thr191’s backbone carboxyl makes contact with the backbone amide N–H of Ala194 and Val195. Proline substitution would not only result in loss of these stabilizing interactions, but also introduce a kink in the alpha-helix. This is expected to induce a conformational change that may disturb the openness of the active site, critical to Polη function ^10^ (Fig. 3).

**Figure 4:**
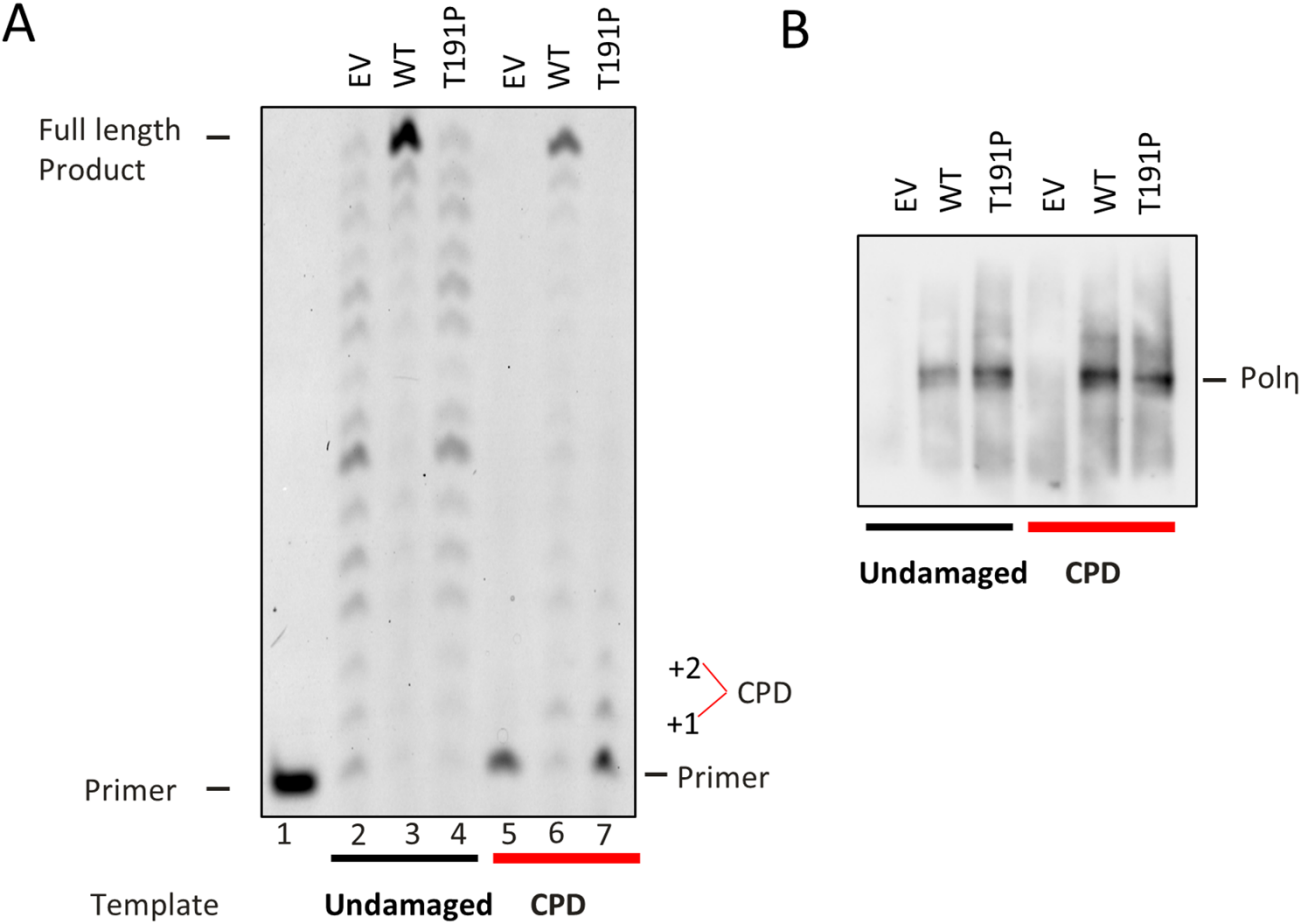
In vitro primer extension assay. (**A**) In vitro transcribed and translated Polη, either wild-type (WT) or T191P, was used to extend an undamaged or a CPD containing template. EV indicates the reticulocyte extract incubated with the Empty Vector. (**B**) Western Blot quantitation of Polη present in the assay.

We therefore sought to characterize this variant by performing a primer extension assay in vitro and by evaluating S-phase stalling and survival in vivo after UV irradiation in stable cell lines expressing wild-type Polη or the Thr191Pro variant. To assess the catalytic properties of the Thr191Pro variant, the substitution was inserted in a Polη coding expression vector that was subsequently used for in vitro coupled transcription and translation in a reticulocyte extract (TnT system, Promega). The variant, along a wild-type (WT) construct and the corresponding empty vector control (EV), was then analyzed in an in vitro primer extension assay using as a 16 bp primer annealed to a 30 bp template, either undamaged or containing a thymidine CPD located in position + 1 respective to the end of the primer (Fig. 4A,B). In the case of the undamaged template, distributive elongation of the primer was observed in the in vitro system, as the reticulocyte extract itself was partially competent for DNA replication (Fig. 4A, lane 2). As previously shown^13^, Polη WT completely elongated the primer in a highly processive manner (Fig. 4A, lane 3). The Thr191Pro variant was comparable to the negative control, already suggesting a deficiency in catalytic activity (Fig. 4A, lane 4). When incubated with a CPD-containing template, the reticulocyte extract was not capable of performing damage bypass and elongating the primer (Fig. 4A, lane 5) resulting in the absence of any longer (> 16 bp) product. TLS activity, on the other hand, might be clearly seen when WT Polη was expressed in the TnT system, where the polymerase was able to extend the primer to completion (Fig. 4A, lane 6). The Thr191Pro substitution renders the polymerase incompetent for bypassing the CPD and incapable of elongating the primer, in a manner again similar to the EV control (Fig. 4A, lane 7).

**Figure 5:**
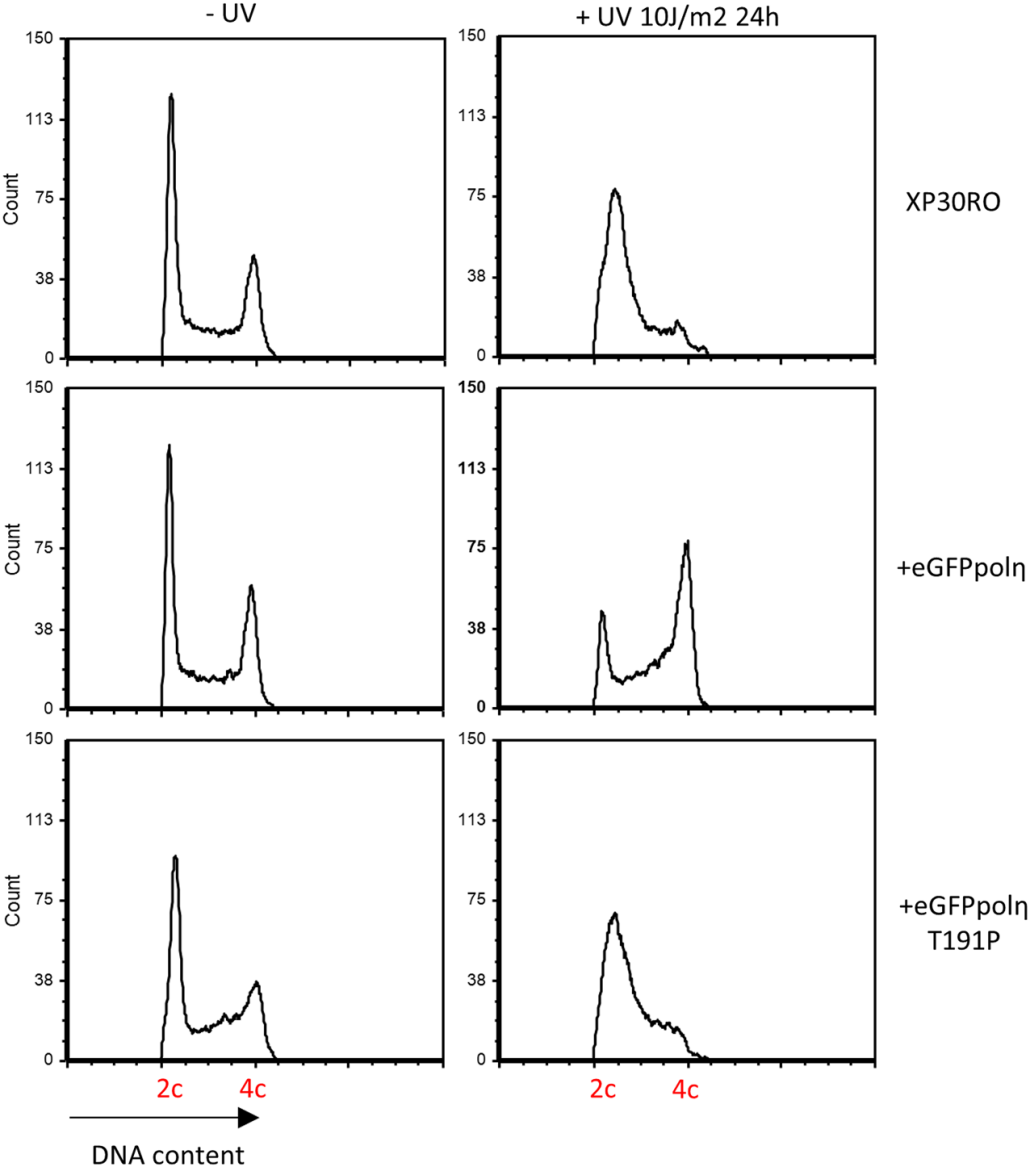
In vivo cell cycle analysis after UV irradiation. Stable cell lines expressing either WT or T191P eGFP-Polη and Polη-deficient XP30RO cells were mock treated or irradiated with 10 J/m^2^ of UV-C and incubated for 24 h. After fixation, the cells were stained with the intercalating agent Propidium Iodide and their DNA content was measured by flow cytometry.

The primer extension assay can minimally assess the polymerase catalytic activity but cannot be considered a substitute for in vivo TLS analysis in cells. In order to obtain further insight into the relevance of the Thr191Pro substitution, we complemented a Polη deficient cell line (XP30RO) with either WT or Thr191Pro Polη, both tagged with eGFP, to assess damage bypass and UV sensitivity. In the first case, cells were irradiated with 10 J/m^2^ and then incubated for 24 h before analyzing their cell cycle distribution by measuring their DNA content via flow cytometry. All three stable cell lines showed comparable cell cycle profiles in undamaged conditions (Fig. 5, left panels), with most of the cells having a peak at 2c DNA content. After UV, Polη deficient cells (XP30RO) could not bypass the damage and arrested in the early stages of S phase. This cell cycle block resulted in a single broad 2c peak (Fig. 5, upper right panel). On the other hand, WT-complemented cells could bypass the damage and progress, albeit slowly, showing an increase in the number of cells in mid to late S phase or even G2 (Fig. 5, middle right panel) with 4c DNA content. As expected from the primer assay result, the Thr191Pro variant behaved essentially as the Polη deficient cell line, indicating its inability to sustain DNA replication in the presence of DNA damage (Fig. 5, lower right panel).

**Figure 6:**
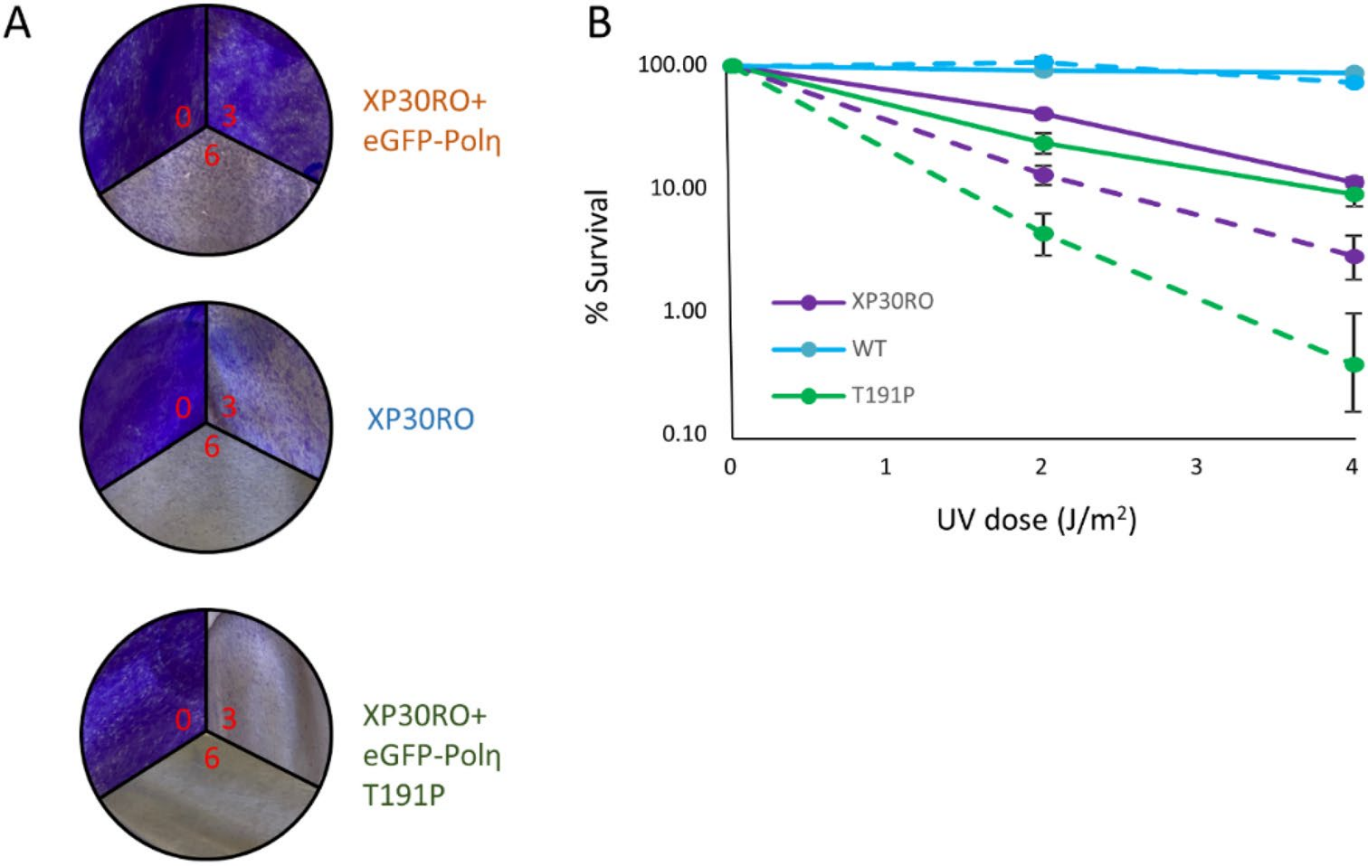
Cell Survival after UV irradiation. (**A**) Composite of representative images of a cell survival assay after increasing doses of UV-C (in red, J/m^2^) following crystal violet staining. The cells lines stably express either WT or T191P eGFP-Polη in a polη deficient background (XP30RO). (**B**) Quantitation of cell survival assays of the same cell lines after crystal violet staining in the absence (solid lines) or in the presence of 75 μg/ml caffeine (dashed lines).

Finally, UV survival assays were carried in the same cell lines. After plating, the cells were exposed to increasing amounts of UV irradiation and incubated in either the absence or the presence of caffeine (Fig. 6A,B). Polη deficient cells show moderate sensitivity to UV (Fig. 6B, compare solid blue and purple lines), that was enhanced by the addition of low doses of caffeine (Fig. 6B, compare dashed blue and purple lines). These results are consistent with the known effects of caffeine on sensitivity to UV-C specifically in this genetic background. In fact, the increased UV sensitivity in the presence of caffeine has been used in the clinic as a diagnostic marker for many years. The cells complemented with WT Polη became resistant to UV-C both in the presence and the absence of caffeine (Fig. 6B, solid and dashed blue lines), whereas the cells complemented with the Thr191Pro variant were even more sensitive to UV irradiation than the parental cells (Fig. 6B, solid and dashed green lines).

## Discussion

DNA polymerase η is a crucial enzyme that allows replication of damaged DNA. Its relevance is exemplified in XPV patients where this polymerase is not functional. Most of the reported mutations are nonsense and occur in the catalytic domain leading to loss of protein function. With our work, we have extensively characterized the previously reported Polη Thr191Pro variant showing that it cannot sustain damage bypass in vitro and impairs S phase progression after UV irradiation in vivo. Finally, the Polη Thr191Pro cannot rescue the sensitivity to UV-C of XPV deficient cells indicating that this allele could be upgraded from a variant of uncertain significance to a likely pathogenic *mutant* according to ACMG guidelines, and as such providing evidence for XPV causality.

Only a small percentage of reported variants are missense, none of which, to this date, has been characterized functionally on a molecular level, thus they are still classified as being of uncertain significance in most databases.

The following overview of naturally occurring *POLH* missense variants attests to that, addressing the structural basis of their putative pathogenicity where predictable. These variants have been reported in the literature with varying phenotypic assessment and different extents of analysis. However, the common approach of using patient-derived cells precludes the criterion ‘strong evidence of pathogenicity’ (PS3) according to ACMG guidelines (Supplementary Table).

The Arg93Pro variant leads to decreased survival in response to UV and caffeine, suggesting abrogation of the UV lesion bypass. This is likely due to the loss of the arginine interaction with the template strand phosphate at the − 2 position, as well as the loss of interaction between the finger and little finger domains, thus undermining the molecular splint that maintains the template strand in the normal B-form in the presence of the UV lesion. Further, the proline is expected to introduce a kink in the alpha helix^14^. Two other arginine missense variants, Arg111His and Arg361Ser, located in the proximity of Arg93, are expected to be analogously damaging to DNA template binding. However, no functional analysis has been done for these two variants. Patients harboring the Arg93Pro variant showed BCC, SCC, and melanoma^14^. Tumoral lesions in patients with Arg111His and Arg361Ser variants were present but not characterized^15^.

Missense variants Gly263Val and Val266Asp, located in the thumb domain, showed no TLS in XPV cell extracts or decreased cell survival after exposure to UV and caffeine. Experiments were done in patient-derived cell lines, thus they do not fulfill functional assay criteria^11,14,15^. Either variant is predicted to result in a less stable protein, but with less severe structure–function defect than Arg93Pro. Tumoral lesions in the respective patients were also present but not characterized.

A palm-domain located Thr122Pro variant was described in the context of compound heterozygosity with a frameshift (His407fsSer442) mutation. XPV but no skin tumors were described for this variant^15^. Another compound heterozygous patient with 24 melanomas and numerous carcinomas had a Gly295Arg variant on one allele, and a Pro576Argfs*3 mutation on another, resulting in truncation after the catalytic core^14^. The long arginine sidechain is likely to result in steric hindrance, thus destabilizing and potentially unfolding the thumb domain.

Three homozygous missense variants, recently identified in XPV patients, are located in the palm (Leu11Pro and Thr191Pro) and little finger (Cys321Phe) domains^12^. The Thr191Pro variant was the only one associated with skin tumors, namely SCC, and is the same missense variant found in our patient. All three variants were reported as of uncertain significance (VUS) in the absence of functional data.

An XPV cell line with a naturally occurring homozygous Ala117Pro missense variant showed slightly decreased unscheduled DNA synthesis and increased UV sensitivity in the presence of caffeine^16^. Similar results were found for an XPV cell line with compound heterozygous variants (Lys535Glu, Lys589Thr)^17^. Another compound heterozygous variant was described with a missense variant on one allele (Lys220Glu) and a frameshift (Ser51Alafs*54) on the other. A translesion synthesis assay was reported to be normal in this cell line, as well as in another with a homozygous Trp174Cys variant^18^. The patients from whom the cell lines were derived developed melanomas in addition to XPV.

It is interesting to note that one missense variant (Met595Val) located in the C-terminal region was not associated with XPV, but rather with melanoma and melanoma-associated carcinomas^19^. There are no functional data available for this variant, but one may speculate that it may be less damaging compared to XPV-associated variants, given the relatively conservative substitution away from the catalytic domain. Indeed, this is an evident example where functional analysis is of utmost importance to determine pathogenicity and an eventual predisposition to non-XPV melanoma.

Another interesting variant (Thr692Ala) was present in an allele harboring a second variant at the stop codon, both at the homozygous state, with the result being a longer (721 aa) than normal (713 aa) protein^20^. Translesion synthesis and UV sensitivity were only slightly affected. The XPV phenotype, which showed no skin tumors, is thought to be due to proteasomal degradation of the exposed UBZ domain. Indeed, the known proteasome inhibitor, bortezomib, restored Polη levels in a cell line stably expressing the longer protein, thus bearing therapeutic potential.

As evident from the above overview, the large majority of naturally occurring missense variants is found in the catalytic domain; only a few were present in the C-terminal of the polymerase. None of the four residues (Cys635, Cys638, His650, His654) that coordinate zinc in the ubiquitin-binding zinc finger (UBZ) domain, or any other UBZ or PCNA-interacting residue, has been found to naturally occur in patients.

To the best of our knowledge, our work describes the first functional analysis of a *POLH* missense variant shown to be catalytically defective in vitro and in vivo, thus confirming XPV causality. While the results are not surprising, based on clinical phenotype and structure–function predictions, it is important to note that without such functional analysis, the Thr191Pro will have otherwise remained VUS. Our data thus contribute to expanding the body of knowledge on Polη and pave the way for characterization of future missense variants, establishing a robust and reliable set of molecular tests that could be quickly implemented to assess variant significance. It will also be interesting to perform functional analysis in variants not associated with XPV, such as Met595Val, or the uncharacterized Thr692Ala variant when present alone and not in the context of a longer protein^21^.

From a clinical perspective, our results enable clear genetic counseling to our patient and her descendants. Further, Polη plays a role in acquired drug resistance through its bypass of lesions induced by platinum-based chemotherapy, and it is a pharmacological target in current major research efforts^3^. Gaining functional insight into critical Polη residues is therefore key to developing small molecule inhibitors for cancer therapeutics.

## Material and methods

### Exome sequencing

Written informed consent was obtained from the patient prior to molecular studies. All methods were carried out in accordance with relevant guidelines and regulations. Whole-exome sequencing was performed on DNA extracted from venous blood, using the Twist Human Core Exome capture kit (Illumina NextSeq500 sequencer), followed by targeted analysis of a 19-gene panel (XP, trichothiodystrophy, or Cockayne syndrome). Reads mapping and variant calling were performed using BWA 0.7.13, Picard 2.9.0 and GATK HaplotypeCaller 3.7 and annotated with annovar 2017-07-17 and UCSC RefSeq (refGene) downloaded on 2018-08-10. Variants were searched for in various databases including Genome Aggregation Database, ClinVar, Leiden Open Variation Database and Human Gene Mutation Database. Written informed consent for publication of clinical and molecular details was obtained from the patient, available on request. The patient was not part of any human experimental study protocol. The *POLH* T191P variant has been deposited in ClinVar under the accession number SCV004218544.

### Cell culture

All the experiments have been carried out with XP30RO (XP-V, also designated GM3617), transformed with SV40 T antigen, carrying a homozygous deletion in the *POLH* gene, resulting in a truncated protein of 42 amino acids. The absence of Polη expression is regularly checked by western blot and the cells are routinely tested for mycoplasma contamination. XP30RO stably expressing Thr191Pro was created as described previously^13^. All the cells were cultured in Dulbecco’s modified Eagle’s medium containing 10% Fetal Bovine Serum.

### UV survival assay

Cell lines expressing different eGFP Polη constructs were plated in triplicate after cell sorting on a Bio-Rad S3e cell sorter. The following day, the cells were washed with PBS, mock treated or irradiated with UV-C (2 and 4 J/m^2^) and incubated in either DMEM or DMEM with 75 μg/ml caffeine. After 72 h the cells were fixed and stained with crystal violet (0.5% Crystal violet in 20% Methanol) for 20 min at RT. After air-drying the plates, the cells were incubated for 20 min at room temperature with destain solution (0.1 M Sodium Citrate pH 4.2 in 25% Ethanol) and the absorbance (@595 nm) of each samples was analysed with a GloMax® Discover Microplate reader.

### Primer extension assay

Primer extension assay was performed as previously described^13^. In brief, plasmids encoding Polη-6His (WT and T191P) were transcribed and translated (TnT, Promega) in vitro in rabbit reticulocyte lysates. Polη protein levels were quantified by western blot analysis and equimolar amounts were used in the primer extension assays. A 5′ FAM-labeled 16mer (5′-CACTGACTGTATGATG-3′) was annealed with a template 30mer primer (5′-CTCGTCAGCATC[cissyn-TT]CATCATACAGTCAGTG-3′) containing a thymidine dimer (TT) at position + 13 (TriLink Biotechnologies) or the undamaged control.

Extension reactions were performed in 10 μl of replication buffer (40 mM Tris–Cl pH 8, 5 mM MgCl_2_, 10 mM DTT, 0.25 mg*/*ml acetylated BSA, 60 mM KCl, 2.5% glycerol) at 37◦C for 15 min before being stopped with of 2 × loading buffer, (98% formamide, 10 mM EDTA pH 8, 0.025% xylene cyanol and 0.025% bromophenol blue). Boiled samples (95◦C for 5 min) were then run on a 15% acrylamide-7 M urea gel and then scanned on a Typhoon TRIO imager (GE HealthCare). Western blots to assess Polη levels were carried out as previously described^22^. Since the blots were used only for assessing Polη, nitrocellulose membranes were cut in the range from 135 to 50 KDa, according to the prestained molecular marker, before incubation with the antibodies (αPolη mAb #13848 Cell Signalling, HRP Goat Anti-Rabbit IgG 111-035-003 Jackson Immunoresearch). Uncropped images are presented in the supplementary files.

### Cell cycle analysis

Cell cycle analysis was performed as in^22^. Briefly, the cells, either mock treated of irradiated with 10 J/m^2^ of UV-C, were incubated for 24 h before fixation in 1 ml of 70% cold ethanol for 4 h. After centrifugation the cells were resuspended in FACS Buffer (PBS, 0.1% Tween 20, 50 μg*/*ml Propidium Iodide, 5 μg*/*ml RNase A) and incubated at 37 °C for 15 min to allow for RNA removal. Finally, the cells were acquired on a Bio-Rad S3e cell sorter and the data was analyzed with FCS Express (De Novo software).

### Supplementary Information

Supplementary Information 1.

Supplementary Information 2.

## Data Availability

All data generated or analysed during this study are included in this published article.
