# Supplementary material for: Homozygous substitution of threonine 191 by proline in polymerase η causes Xeroderma pigmentosum variant
Source: Sci Rep. 2024 Jan 11;14:1117. doi: 10.1038/s41598-023-51120-1 (PMC10784498; doi:10.1038/s41598-023-51120-1)
Supplement: Supplementary file 1 — Supplementary Information 1. [file 41598_2023_51120_MOESM1_ESM.docx]

| **Polη variant** | **Zygosity** | **Domain** | **XPV** | **Tumor type** | **Damage Bypass in XPV cell lines** | **Reference** |
| --- | --- | --- | --- | --- | --- | --- |
| **Arg93Pro** | Homozygous | Palm (DNA-binding) | Yes | BCC, SCC, melanoma | No | [14] |
| **Arg111His** | Homozygous | Palm (DNA-binding) | Yes | Not characterized | Not characterized | [15] |
| **Arg361Ser** | Homozygous | Little finger | Yes | Not characterized | Not characterized | [15] |
| **Gly263Val** | Homozygous | Thumb | Yes | Not characterized | No | [14] |
| **Val266Asp** | Homozygous | Thumb | Yes | Not characterized | No | [15] |
| **Thr122Pro** | Compound heterozygous with frameshift | Palm | Yes | Not characterized | Not characterized | [15] |
| **Gly295Arg** | Compound heterozygous with frameshift | Thumb | Yes | BCC, SCC, melanoma | Not characterized | [14] |
| **Leu11Pro** | Homozygous | Palm | Yes | None | Not characterized | [12] |
| **Thr191Pro** | Homozygous | Palm | Yes | BCC, SCC, melanoma | Not characterized | [12] and this study |
| **Cys321Phe** | Homozygous | Little finger | Yes | None | Not characterized | [12] |
| **Ala117Pro** | Homozygous | Palm | Yes | melanoma | No | [16] |
| **Lys535Glu** | Compound heterozygous with Lys589Thr | C-terminal | Yes | None | No | [17] |
| **Lys220Glu** | Compound heterozygous with frameshift | Palm | Yes | BCC, SCC, melanoma | Yes | [18] |
| **Trp174Cys** | Homozygous | Palm | Yes | melanoma | Yes | [18] |
| **Met595Val** | Heterozygous | C-terminal | No | melanoma | Not characterized | [19] |
| **Thr692Ala** | Homozygous in longer protein with stop codon variant | C-terminal | Yes | None | No | [20] |

**Supplementary Table:** Overview of the major naturally occurring Polη missense variants, for which no functional characterization has been done, except for Thr191Pro in the present study (adapted from [23]).
