# Supplementary material for: Homozygous substitution of threonine 191 by proline in polymerase η causes Xeroderma pigmentosum variant
Source: Sci Rep. 2024 Jan 11;14:1117. doi: 10.1038/s41598-023-51120-1 (PMC10784498; doi:10.1038/s41598-023-51120-1)
Supplement: Supplementary file 2 — Supplementary Information 2. [file 41598_2023_51120_MOESM2_ESM.pdf]

A

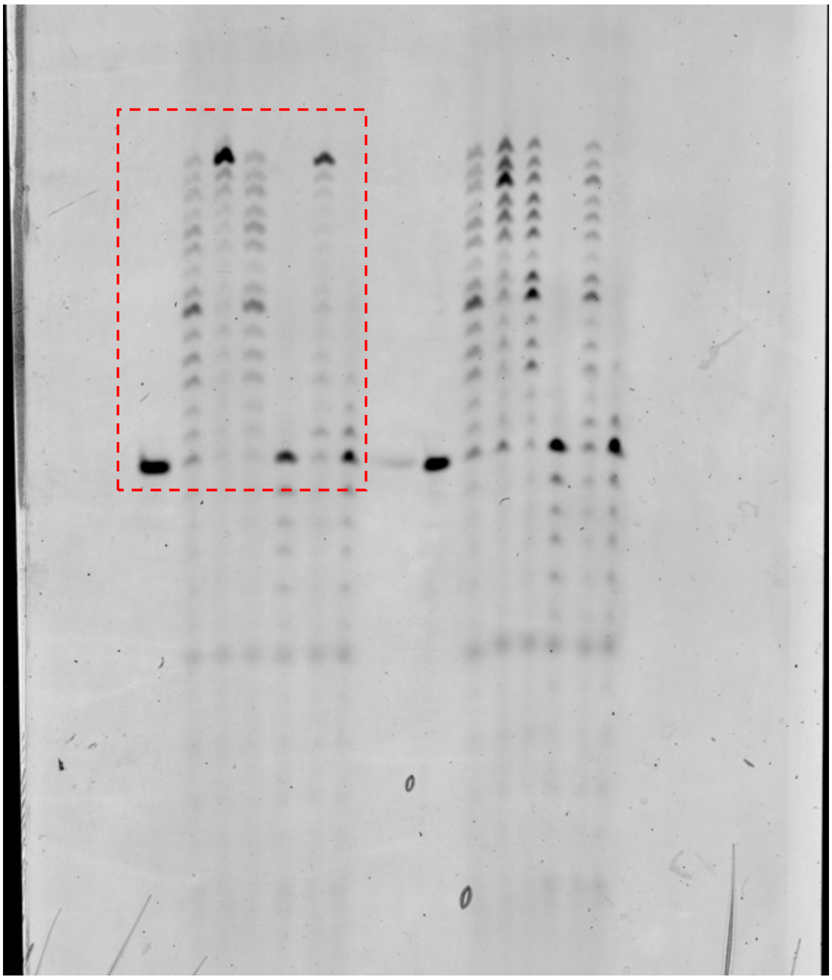

B

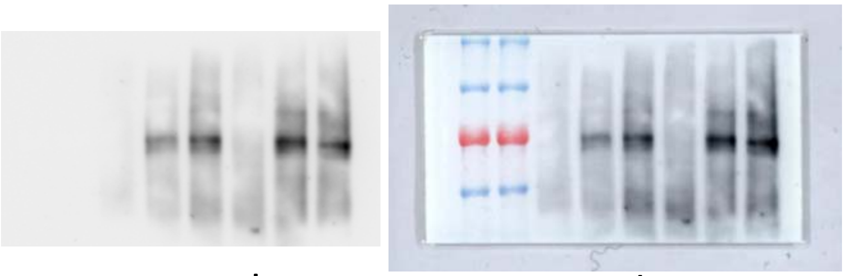

Supplementary Figure 1. Uncropped scan of Fig4. (A) Denaturing sequencing gel used in the primer extension assay. (B) Primer extension assay samples loaded on SDS-PAGE to assess the amount of polη (left: raw chemiluminescence, right: overlay with protein marker).

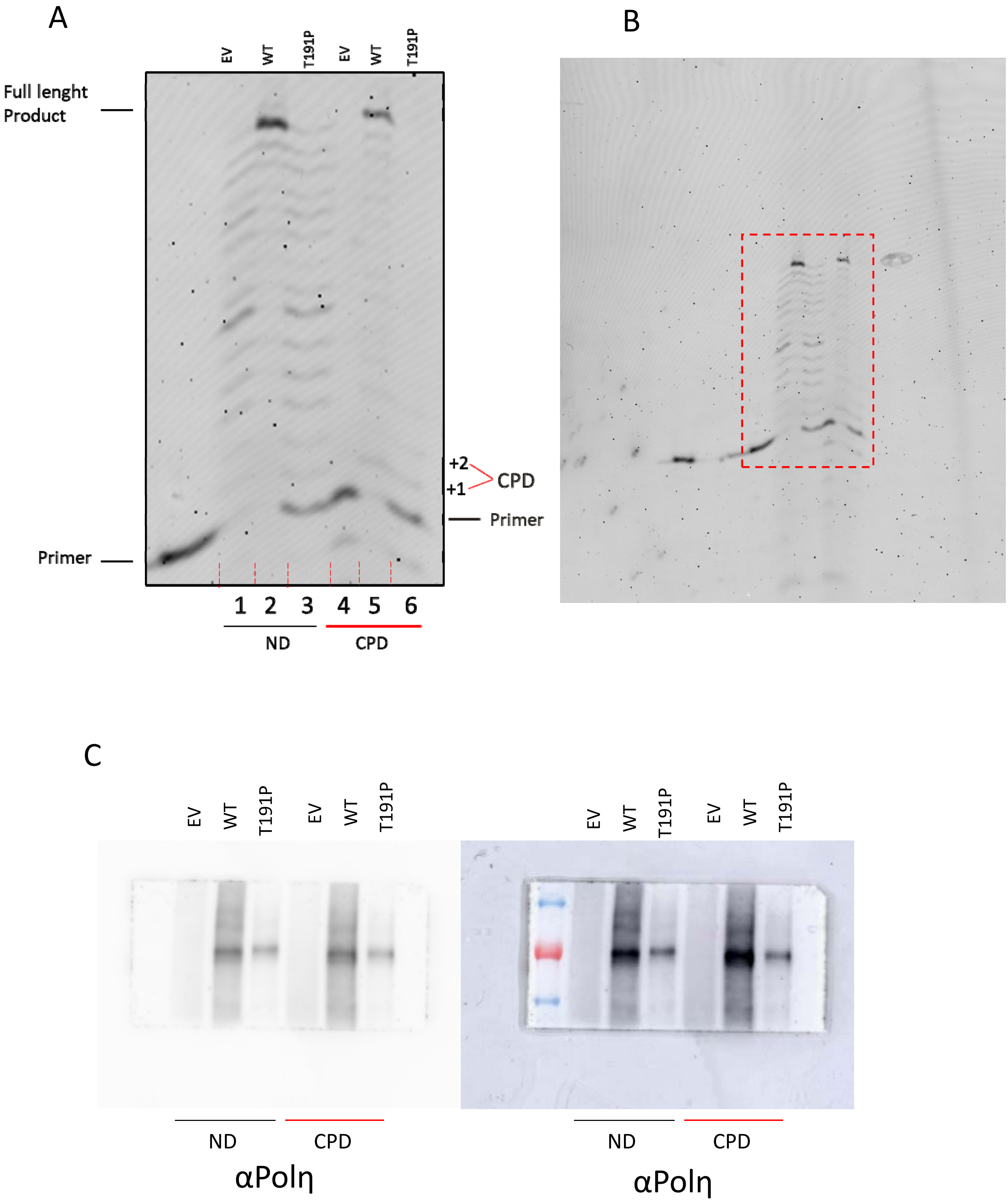

Supplementary Figure 2. Replicate of the experiment shown in Fig4 and Supplementary Figure1. (A) Denaturing sequencing gel used in the primer extension assay. Dotted line indicates lanes width. (B) Uncropped scan of the primer extension assay. The dotted box indicates the part of the scan used in A. (C) Primer extension assay samples loaded on SDS-PAGE to assess the amount of polη (left: raw chemiluminescence, right: overlay with protein marker).
